# Hypoglycaemic stimulation of macrophage cytokine release is suppressed by AMP‐activated protein kinase activation

**DOI:** 10.1111/dme.15456

**Published:** 2024-12-24

**Authors:** Jiping Zhang, Alice E. Pollard, Eleanor F. Pearson, David Carling, Benoit Viollet, Kate L. J. Ellacott, Craig Beall

**Affiliations:** ^1^ Department of Clinical and Biomedical Sciences, Faculty of Health and Life Sciences University of Exeter Medical School Exeter UK; ^2^ MRC London Institute of Medical Sciences, Imperial College London Hammersmith Hospital London UK; ^3^ Institute Cochin Université Paris Cité, CNRS, Inserm Paris France

**Keywords:** AMP‐activated protein kinase, hypoglycaemia, inflammation, macrophage, macrophage migration inhibitory factor, PF‐06409577

## Abstract

**Aims:**

Acute hypoglycaemia promotes pro‐inflammatory cytokine production, increasing the risk for cardiovascular events in diabetes. AMP‐activated protein kinase (AMPK) is regulated by and influences the production of pro‐inflammatory cytokines. We sought to examine the mechanistic role of AMPK in low glucose‐induced changes in the pro‐inflammatory cytokine macrophage migration inhibitory factor (MIF), which is elevated in people with diabetes.

**Methods:**

Macrophage cell line Raw264.7 cells, primary macrophage bone marrow‐derived macrophages obtained from wild‐type mice or AMPK γ1 gain‐of‐function mice, were used, as were AMPKα1/α2 knockout mouse embryonic fibroblasts (MEFs). Allosteric AMPK activators PF‐06409577 and BI‐9774 were used in conjunction with inhibitor SBI‐0206965. We examined changes in protein phosphorylation/expression using western blotting and protein localisation using immunofluorescence. Metabolic function was assessed using extracellular flux analyses and luciferase‐based ATP assay. Cytokine release was quantified by enzyme‐linked immunosorbent assay (ELISA). Oxidative stress was detected using a fluorescence‐based reactive oxygen species (ROS) assay, and cell viability was examined using flow cytometry.

**Results:**

Macrophages exposed to low glucose showed a transient and modest activation of AMPK and a metabolic shift towards increased oxidative phosphorylation. Moreover, low glucose increased oxidative stress and augmented the release of macrophage MIF. However, pharmacological activation of AMPK by PF‐06409577 and BI‐9774 attenuated low glucose‐induced MIF release, with a similar trend noted with genetic activation using AMPKγ1 gain‐of‐function (D316A) mice, which produced a mild effect on low glucose‐induced MIF release. Inhibition of NFĸB signalling diminished MIF release and AMPK activation modestly but significantly reduced low glucose‐induced nuclear translocation of NFĸB.

**Conclusions:**

Taken together, these data indicate that pharmacological AMPK activation suppresses the release of MIF from macrophages caused by energy stress, suggesting that AMPK activation could be a useful strategy for mitigating hypoglycaemia‐induced inflammation.

## Graphical abstract

Low glucose induces pro‐inflammatory MIF release from macrophages, and pharmacological AMPK activation suppresses the release of MIF. The AMPK/NFĸB signalling pathway is involved, which may be a new strategy to attenuate the pro‐inflammatory response in hypoglycaemia.

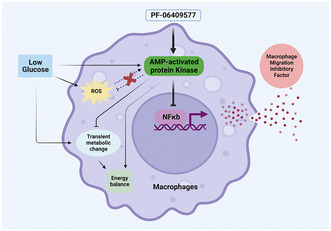

**What‘s new?**

**What is already known?**

Hypoglycaemia induces inflammation AMP‐activated protein kinase (AMPK) senses cellular energy stress and has an anti‐inflammatory role in many tissues.

**What this study found?**

Low glucose increased macrophage migration inhibitory factor (MIF) release from macrophages This was linked to low glucose‐induced oxidative stress Pharmacological activation of AMPK attenuated low glucose induced MIF release.

**What are the implications of this study?**

AMPK activators could be used to attenuate the pro‐inflammatory response in hypoglycaemia.

## 1 INTRODUCTION

Chronic low‐grade inflammation contributes to the pathogenesis of diabetes.^1^ People with diabetes have higher circulating levels of inflammatory markers, including macrophage migration inhibitor (MIF),^2^ C‐reactive protein (CRP), interleukin‐6 (IL‐6) and tumour necrosis factor‐α (TNF‐α).^3^ MIF is a cytokine expressed in the outer lining of tissues such as endothelial, epithelial, endocrine and immune cells, including macrophages and can be released in response to cellular stress from readily releasable pools.^4^ MIF is also highly expressed in diabetes complications such as myocardial damage,^5^ coronary artery disease and diabetic retinopathy,^6^ suggesting a graded increase in circulating MIF with disease severity. The cytokine increases the production of IL‐1β, IL‐6 and TNFα^4^ and may also play a role in thrombosis.^7^ In contrast, MIF deficiency^8^ or antagonism^9^ has been shown to prevent chronic inflammation, glucose intolerance and insulin resistance in mice, suggesting that MIF plays a detrimental role in diabetes.

Recent studies have demonstrated that hypoglycaemia in addition to hyperglycaemia contributes to inflammation via increasing circulating markers such as CRP and oxidative stress.^10^ Hypoglycaemia also increases the levels of IL‐1β, IL‐6 and TNFα,^11^ similarly to MIF. Importantly, hypoglycaemia increases the risk for a cardiovascular event^12^ and is also associated with neurological damage and increased risk for mortality.^13^ As a major source of cytokines, including MIF, macrophages play an important role in chronic inflammation in diabetes. The macrophage inflammatory response is an energy‐intense process, with metabolism shifting from mainly oxidative metabolism to aerobic glycolysis during the pro‐inflammatory phase.^14^ This switch is associated with reduced activation of the key energy sensor adenosine monophosphate‐activated protein kinase (AMPK) activation, which can be blocked by pharmacological activation of AMPK.^15^ The kinase senses the nucleotide and acts to restore energy balance by inhibiting anabolic, while promoting catabolic processes.^16^ The first‐line Type 2 diabetes drug metformin, which also activates AMPK,^17^ reduces systemic inflammation by decreasing the level of CRP and IL‐6^18^ and lowers plasma macrophage migration in obese patients.^15^ Here, we hypothesised that hypoglycaemic levels of glucose would increase MIF release from macrophages and in a manner suppressed by pharmacological AMPK activation. We used AMPK activators, PF‐06409577,^19^ given its use in a recent phase I clinical study (NCT02286882), and BI‐9774, in conjunction with AMPK gain‐of‐function mice, to examine whether AMPK activation could prevent or reduce pro‐inflammatory MIF production from macrophages following hypoglycaemia‐like stress.

## 2 MATERIALS AND METHODS

### 2.1 Cell isolation and culture

Raw264.7 cells and primary BMDMs (bone marrow derived marcophages) were cultured as previously described.^20^ Bone marrow was collected from C57BL/6J wild‐type healthy mice. Immediately before treatments, cells were cultured in serum‐free DMEM (Dulbecco's Modified Eagle Medium) or RPMI medium with 5.5 mmol/L glucose and then transferred to experimental conditions. AMPKγ1 gain‐of‐function mice (*prkag1*, aspartic acid to alanine at residue 316; [D316]) were generated as described previously,^21^ and BMDMs were isolated from male wild‐type (WT) or AMPKγ1 D316A mutant mice. Mouse embryonic fibroblasts (MEFs) WT and AMPK α1/2 (−/−) double knockout cells were generated as previously described^22^ and were cultured as described before.^23^

### 2.2 Animals

Mice (male) were housed in pathogen‐free individually ventilated cages on 12 hr light/dark cycles and maintained at a constant temperature of 22 ± 1°C. Mice were group housed (up to 4 per cage) and *ab libitum* fed a standard diet (Exeter: 2018, Teklad Global; aged 9–11 weeks; Imperial: RM3 diet, Special Diet Services; aged 11–14 months [WT and AMPKγ1 D316A mice]) with free access to water. Animals were provided woodchip bedding, tissue nesting paper with environmental enrichment. Animals were culled by Schedule 1 method and all studies were performed in accordance with the United Kingdom Animals (Specific Procedures) Act (1986) and approved by the Animal Welfare and Ethical Review Boards at the University of Exeter and Imperial College London.

### 2.3 Western blotting

Cellular protein was isolated as previously described.^24^ Briefly, 2.5 × 10^6^ Raw264.7 cells were seeded onto 60 mm Petri dishes 1 day before treatment and harvested with lysis buffer. BMDMs were seeded onto 60 mm petri dishes 1 day before treatment. Protein concentrations were assessed by the method of Bradford. Ten‐microgram protein of each sample was loaded on 7%, 10% or 15% (wt/vol.) polyacrylamide gels, transferred to nitrocellulose membranes, blocked with powdered milk or bovine serum albumin (2.5–5% wt/vol.) in TBS/T and probed with antibodies against target proteins. Images were captured by infrared imaging, and changes in expression were quantified by densitometry. Changes in phosphorylation were normalised to total expression of the target protein.

### 2.4 Immunocytochemistry

Immunocytochemical staining was performed as previously described.^25^ Briefly, cells were plated on 13 mm diameter glass coverslips. After treatment, cells were fixed, permeabilised and non‐specific binding blocked using donkey serum before incubating with mouse anti‐NFĸBp65 antibody (1:1000 in TBST; overnight at 4°C) and secondary donkey anti‐mouse Alexa fluor488 (1:500 in PBS‐T) for 1 h at room temperature. Coverslips were mounted in DAPI‐containing mounting media (ab104139, Abcam, Cambridge, UK) before imaging using a Leica SP8 confocal microscope. FIJI software was used to manually analyse fluorescent staining. Nuclear/cytoplasmic ratios were calculated as follows: nuclear mean intensity/[(cell mean intensity × cell area − nucleus mean intensity × nucleus area)/(cell area − nucleus area)] × 100.

### 2.5 Enzyme‐linked immunosorbent assay (ELISA)

Cytokines secreted into culture supernatants were measured by ELISA according to the manufacturers' protocols (DY1978, Biotechne, Abingdon, UK). Briefly, media supernatants were collected and diluted, as required. Extracellular cytokine levels were normalised to total protein, unless otherwise stated.

### 2.6 Measurement of ATP levels

ATP levels were measured using ATPlite assay kit (6016941, Perkin Elmer, Seer Green, UK) as previously described.^23^ For extracellular ATP, 100 μL of media supernatant was used per sample, in black‐walled 96‐well plates. The luminescence was read using the PheraStar microplate reader (BMG LabTech). ATP concentrations were calculated and normalised to total protein.

### 2.7 Measurement of cellular metabolism and viability

Raw 264.7 cells and BMDMs were seeded in Agilent XFe96 culture plates (102416–100, Agilent, Craven Arms, UK) 24 h prior to treatment. Metabolic measurements were performed following the manufacturer's instructions. Briefly, the medium was exchanged with DMEM or RPMI Seahorse XF medium pH 7.4 and cells were degassed for 1 hr. Extracellular acidification rate (ECAR) as a measure of glycolysis, and oxygen consumption rate (OCR) as a measure of mitochondrial function normalised to the total protein content. Cell viability assay was performed by propidium iodide (PI) (P4864, Merck, Gillingham, UK) staining followed by flowcytometry analysis.^25^ See ESM for further details.

### 2.8 Reactive oxygen species (ROS) measurement

Raw 264.7 cells were cultured in 48‐well plates. Following treatments, cells were loaded with 10 μmol/L of DCFDA (D399; Invitrogen, Loughborough, UK) in DMEM (6‐carboxy‐2',7'‐dichlorodihydrofluorescein diacetate) and incubated at 37°C for 30 min. The levels of ROS were measured using PheraStar microplate reader. Relative fluorescence units were normalised to the total protein content.

### 2.9 Statistical analysis

Two‐group comparisons were determined by t‐test using GraphPad Prism software (Prism 9; GraphPad Software, La Jolla, CA, USA). For normalised immunoblotting data, a one sample t‐test in comparison to control was used. For multiple group comparisons, a one‐way ANOVA with Bonferroni statistical tests was performed. To compare the mean differences between groups split by two or three independent variables, a two‐way ANOVA or a three‐way ANOVA with Bonferroni multiple comparisons test was used, respectively. Results are expressed as mean ± standard error. Values of *p* < 0.05 were considered statistically significant.

## 3 RESULTS

### 3.1 Activation of AMPK in macrophages

**FIGURE 1:**
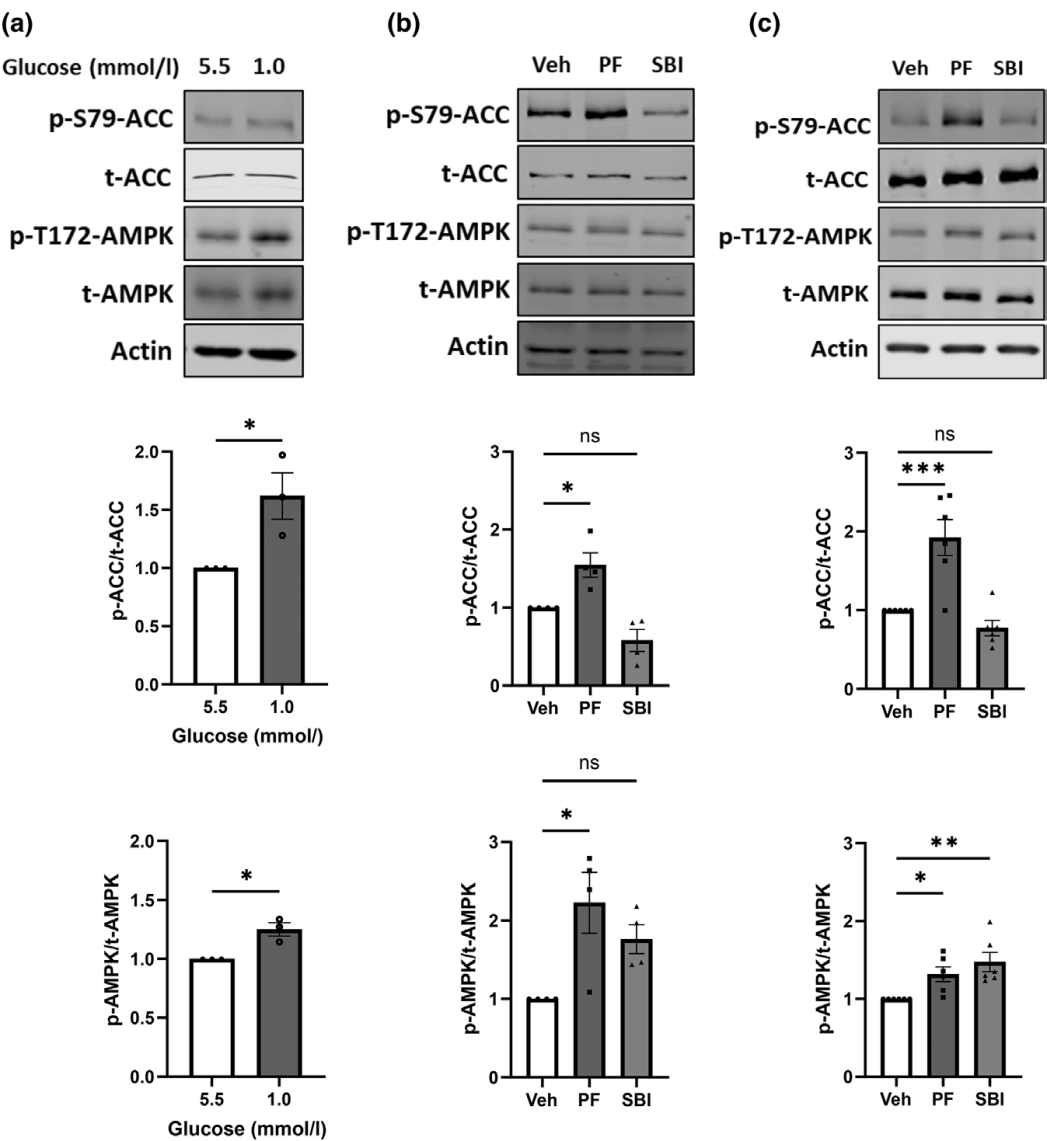
Low glucose and PF‐06409577 increased phosphorylation of AMPK in macrophages. (a) Representative immunoblots of Raw264.7 cells cultured with either 5.5 mmol/L or 1.0 mmol/L glucose for 30 min (*n* = 3). (b) Representative immunoblots of Raw264.7 exposed to vehicle (0.1% v/v DMSO) (Veh), 10 μmol/L of PF‐06409577 (PF) or 30 μmol/L of SBI‐0206965 (SBI) for 24 h (*n* = 4). (c) Representative immunoblots of BMDMs exposed to vehicle (0.1% v/v DMSO) (Veh), 10 μmol/L of PF‐06409577 (PF) or 30 μmol/L of SBI‐0206965 (SBI) for 16 h (*n* = 6). Cells were then lysed and immunoblots were prepared. Densitometric analysis of immunostaining for phosphorylated protein was normalised to total protein level. p‐ACC, phospho‐acetyl‐CoA carboxylase (S79) (p‐S79‐ACC); p‐AMPK, phospho‐AMP‐activated protein kinase (T172) (p‐T172‐AMPK); t‐ACC, total acetyl‐CoA carboxylase; t‐AMPK, total AMP‐activated protein kinase. Data are expressed as mean ± SEM. Comparisons between groups were made by unpaired t‐test (a) or one‐way ANOVA with Bonferroni's multiple comparisons test (b, c). *p < 0.05; **p < 0.01; ***p < 0.001; ns, not significantly different.

To examine whether low glucose activates AMPK in macrophages, we exposed Raw264.7 cells to normal (5.5 mmol/L) and low (1.0 mmol/L) glucose levels for 30 min. This increased phosphorylation of AMPK at threonine 172 (T172), a site required for full kinase activation (Figure 1a). Phosphorylation of acetyl‐CoA carboxylase (ACC, ser79), a downstream substrate of AMPK, was also increased (Figure 1a), indicating increased AMPK activity. However, after 16 h of low glucose, neither the level of AMPK or ACC phosphorylation was altered in either Raw264.7 cells or BMDMs (ESM Figure 1).

We next examined the effect of pharmacological AMPK manipulation using activator PF‐06409577 (10 μmol/L) and AMPK inhibitor SBI‐0206965 (30 μmol/L) on AMPK pathway activity in Raw264.7 and BMDMs. PF‐06409577 increased phosphorylation of AMPK and ACC in both macrophage cell types (Figure 1b,c). Consistent with a previous report,^26^ SBI‐0206965 increased AMPK phosphorylation at T172 yet showed a trend towards decreased p‐ACC in both Raw264.7 cells and BMDMs (Figure 1 b,c), indicating AMPK inhibition, as expected. Metformin at relatively high concentrations (250 μmol/L) did not sufficiently activate AMPK in macrophages and did not alter low glucose‐induced MIF release (ESM Figure 2).

**FIGURE 2:**
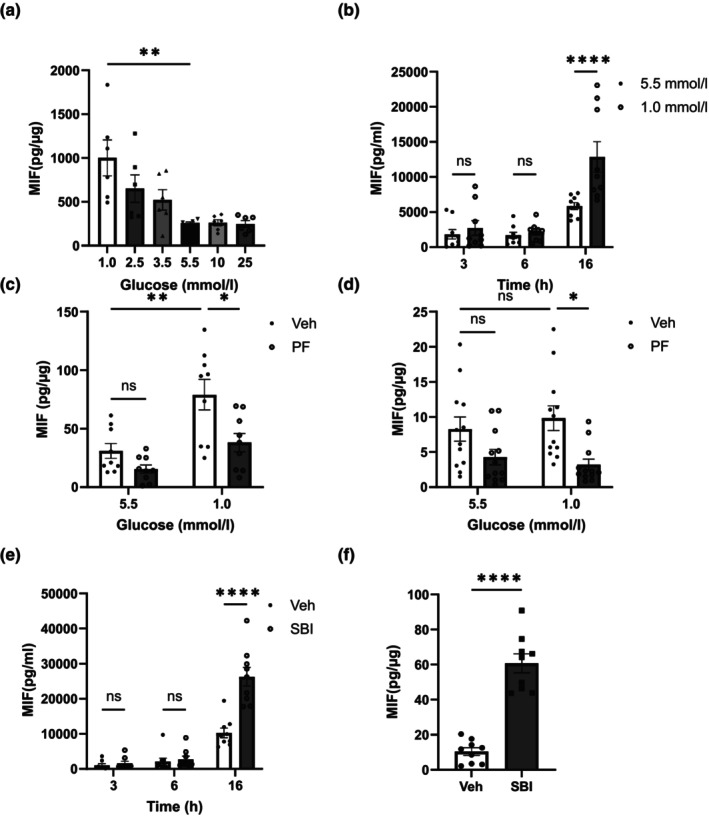
Low glucose (1.0 mmol/L) increased MIF release from Raw264.7 cells, and MIF release was attenuated by PF‐06409577 in Raw264.7 cells and BMDMs, but SBI‐0206965 enhanced MIF release in both cell types. (a) Raw264.7 cells were cultured with different concentrations of glucose (1.0, 2.5, 3.5, 5.5, 10 and 25 mmol/L) for 24 h (*n* = 6). (b) Raw264.7 cells were cultured with normal glucose (5.5 mmol/L) or low glucose (1.0 mmol/L) for 3, 6 and 16 h (*n* = 9). (c) Raw264.7 cells (*n* = 9) and (d) BMDMs were cultured with normal glucose (5.5 mmol/L) or low glucose (1.0 mmol/L) in the presence of vehicle (0.1% v/v DMSO) (Veh) or 10 μmol/L PF‐06409577 (PF) for 16 h (*n* = 12). (e) Raw264.7 cells (*n* = 9) and (f) BMDMs were cultured with normal glucose levels (5.5 mmol/L) in the presence of vehicle (0.1% v/v DMSO) (Veh) or 30 μmol/L SBI‐0206965 (SBI) for 16 h (*n* = 9). The MIF level in the medium was assessed by ELISA. The amount of released MIF was shown as concentrations (pmol/ml) (b, e), or normalised to the total protein amount in each sample (a, c, d, f). Data are expressed as mean ± SEM. Comparisons between groups were made by unpaired t‐test (f), one‐way ANOVA (a) or two‐way ANOVA with Bonferroni's multiple comparisons test (b, c, d, e). *p < 0.05; **p < 0.01; ****p < 0.0001; ns, not significantly different.

To examine the consequence of glucose variation on MIF release from macrophages, Raw264.7 cells were cultured in the presence of decreasing concentrations of glucose. Low glucose (1 mmol/L, 2.5 mmol/L, 3.5 mmol/L) induced MIF release in a concentration‐responsive manner from Raw264.7 cells, compared with normal glucose (5.5 mmol/L; Figure 2a,b). In contrast, high glucose (>10 mmol/L) did not alter MIF release from Raw264.7 cells (Figure 2a). These differences could not be accounted for by a change in cell viability (ESM Figure 3a).

**FIGURE 3:**
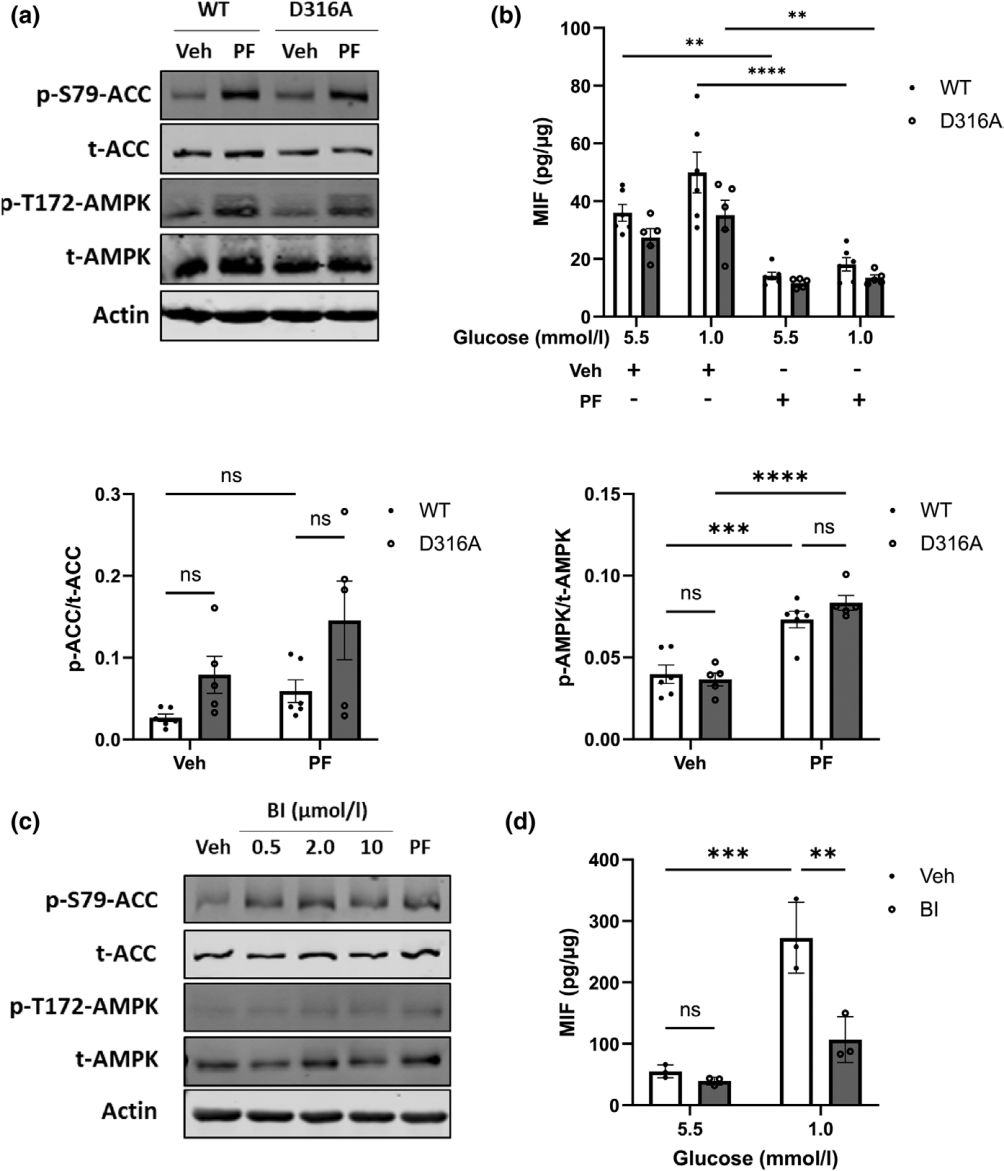
PF‐06409577 activated AMPK and inhibited MIF release from both wild‐type and D316A BMDM and low glucose‐induced MIF release from Raw264.7 cells was attenuated by BI‐9774. (a) Representative immunoblots of BMDM exposed to vehicle (0.1% v/v DMSO) (Veh) or 10 μmol/L of PF‐06409577 (PF) for 16 h (WT, *n* = 6; D316A, *n* = 5). BMDM cells were obtained from either wild‐type (WT) or AMPKγ1 D316A (D316A) mice. Densitometric analysis of immunostaining for phosphorylated protein was normalised to total protein level. P‐ACC, phospho‐acetyl‐CoA carboxylase (S79) (p‐S79‐ACC); p‐AMPK, phospho‐AMP‐activated protein kinase (T172) (p‐T172‐AMPK); t‐ACC, total acetyl‐CoA carboxylase; tAMPK, total AMP‐activated protein kinase. (b) BMDM cells obtained from either wild‐type (WT) or D316A mice (D316A) were cultured with normal level of glucose (5.5 mmol/L) or low level of glucose (1.0 mmol/L) in the presence of vehicle (0.1% v/v DMSO) (Veh) or PF‐06409577 (PF) for 16 h. The MIF release in the medium was assessed by using ELISA (WT, *n* = 6; D316A, *n* = 5). (c) Raw264.7 cells were exposed to vehicle (0.1% v/v DMSO) (Veh) or different concentrations (0.5, 2.0 and 10 μmol/L) of BI‐9774 (BI) or 10 μmol/L PF‐06409577 (PF) for 16 h, and then cells were lysed and immunoblots were prepared (*n* = 1). (d) Raw264.7 cells were cultured with normal level of glucose (5.5 mmol/L) or low level of glucose (1.0 mmol/L) in the presence of vehicle (0.1% v/v DMSO) (Veh) or 0.5 μmol/L of BI‐9774 (BI) for 16 h. The MIF level in the medium was assessed by ELISA. The amount of released MIF was normalised to the total protein amount in each sample (*n* = 3). Data are expressed as mean ± SEM. Comparisons between groups were made by two‐way ANOVA (a, d) or three‐way ANOVA (b) with Bonferroni's multiple comparisons test. **p < 0.01; ***p < 0.001; ****p < 0.0001; ns, not significantly different.

To determine whether AMPK plays a role in MIF release from macrophages, Raw264.7 and mouse primary BMDMs were exposed to AMPK activators in normal and low glucose (1.0 mmol/L) levels. PF‐06409577 attenuated MIF release in both Raw264.7 cells and BMDMs (Figure 2c,d), which was replicated by chemically distinct AMPK activator BI‐9774^27^ (Figure 3d). This change in MIF release was not accompanied by a change in cellular MIF expression (ESM Figure 4).

To examine whether genetic activation of AMPK alters MIF release, BMDMs were obtained from WT or AMPKγ1 transgenic mice (hereafter, referred to as AMPK D316A mice), which display elevated basal AMPK activity as previously described.^21^ ACC phosphorylation was modestly increased in BMDMs isolated from AMPK D316A mice, although not significantly (Figure 3a). MIF release modestly but not significantly decreased in BMDMs isolated from AMPK D316A (Figure 3b). PF‐06409577 further decreased the MIF release at both normal glucose (5.5 mmol/L) and low glucose (1.0 mmol/L) levels (Figure 3b).

### 3.2 AMPK activation prevented low glucose‐induced changes in cellular metabolism

**FIGURE 4:**
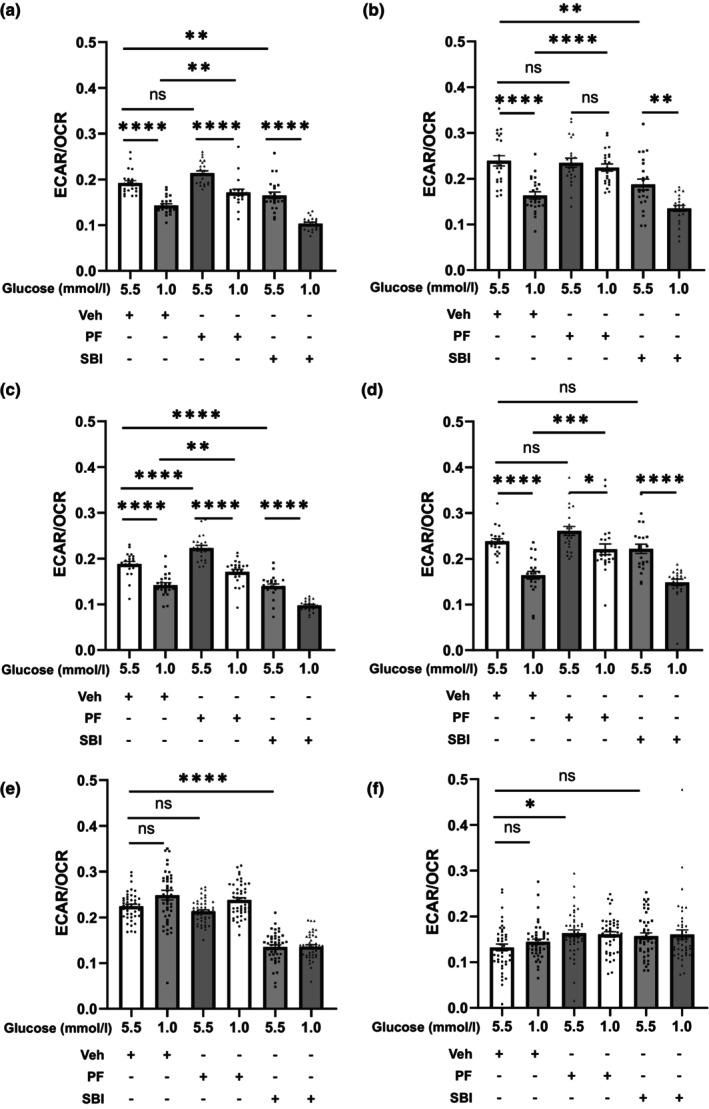
Low glucose reduced the ratio of glycolysis to oxidative phosphorylation, and PF‐06409577 restored the ratio in Raw264.7 cells and BMDM at 1 h and 3 h but not at 16 h. (a) Raw264.7 cells and (b) BMDM were cultured in the presence of different concentrations of glucose (5.5 mmol/L and 1.0 mol/L) with vehicle (0.1% v/v DMSO) (Veh) or 10 μmol/L PF‐06409577 (PF) or 30 μmol/L SBI‐0206965 (SBI) for 1 h (a, b), 3 h (c, d) or 16 h (e, f) in Seahorse XF96 cell culture microplates. Then, the contribution of glycolysis over oxidative phosphorylation was expressed as the ratio of extracellular acidification rate (ECAR) to oxygen consumption rate (OCR) using the Seahorse BioAnalyzer XFe96. Data are expressed as mean ± SEM (a–d, *n* = 23; e, f, *n* = 45 or 48). Comparisons between groups were made by two‐way ANOVA Bonferroni's multiple comparisons test. *p < 0.05; **p < 0.01; ***p < 0.001; ****p < 0.0001; ns, not significantly different.

Raw264.7 cells and BMDMs exposed to low glucose had a decreased ECAR/OCR ratio up to 3 h (Figure 4a–d), which was not apparent at 16 h (Figure 4e,f). The effect was largely driven by a reduction in ECAR with a more modest increase in OCR. Interestingly, PF‐06409577 prevented the low glucose‐induced reduction ECAR/OCR ratio to levels comparable to control. Despite the significant reduction in glucose availability, we found that intracellular levels of ATP were well defended over the time course, with no significant changes observed during low glucose exposure or with PF‐06409577 (ESM Figure 5).

### 3.3 Low glucose‐induced oxidative stress

**FIGURE 5:**
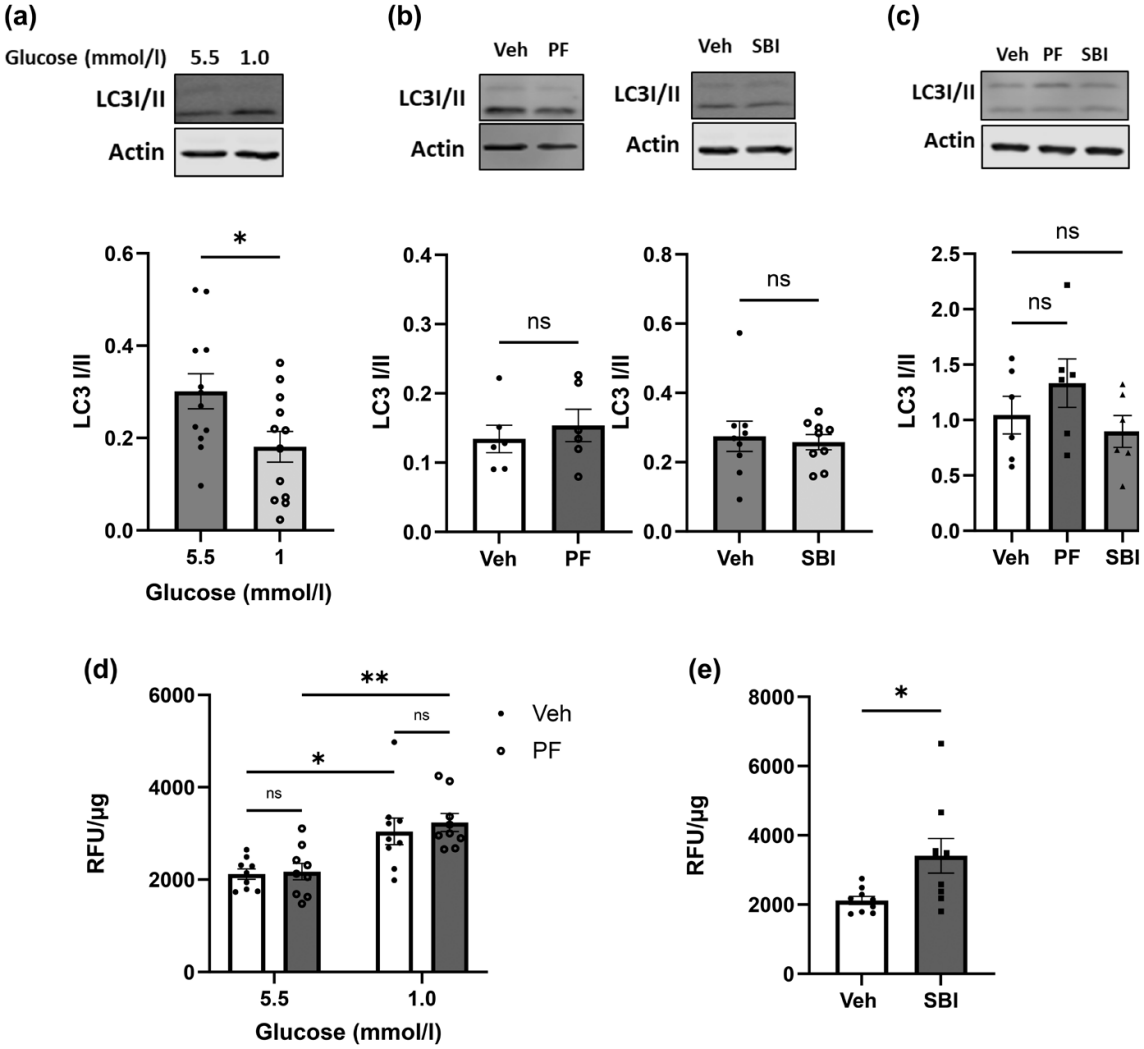
Low glucose activated autophagy and elevated ROS level, but PF‐06409577 did not alter autophagy or ROS level in Raw264.7 cells. **(**a) Representative immunoblots of Raw264.7 cultured with either 5.5 mmol/L or 1.0 mmol/L glucose for 16 h (*n* = 12). (b) Representative immunoblots of Raw264.7 cells exposed to vehicle (0.1% v/v DMSO) (Veh) or PF‐06409577 (PF; *n* = 6) or SBI‐0206965 (SBI; *n* = 9) for 16 h. (c) Representative immunoblots of BMDMs exposed to vehicle (0.1% v/v DMSO) (Veh) or PF‐06409577 (PF) or SBI‐0206965 (SBI) for 16 h (*n* = 6). Densitometric analysis of immunostaining for LC 3 I and LC3 II. The ratio of LC3 I/II demonstrates the activity of autophagy. (d) ROS assay results of Raw264.7 cells cultured in the presence of 5.5 mmol/L or 1.0 mmol/L glucose with vehicle (0.1% v/v DMSO) (Veh) or PF‐06409577 (PF) for 16 h (*n* = 9). (e) ROS assay results of Raw264.7 cells exposed to vehicle (0.1% v/v DMSO) (Veh) or SBI‐0206965 (SBI) for 16 h. Following by staining with DCFDA and assessed in PheraStar plate reader and normalised to total protein content in the same well (*n* = 9). Data are expressed as mean ± SEM. Comparisons between groups were made by t‐test (a, b, e) or by one‐way ANOVA (c) or by two‐way ANOVA with Bonferroni's multiple comparisons test (d). *p < 0.05; **p < 0.01; ns, not significantly different.

We next probed for changes in autophagy given that both low glucose/AMPK activation can promote autophagy by phosphorylating ULK1 and mTOR.^28^ Autophagy activation by low glucose was noted by a significantly decreased ratio of LC3I/II (Figure 5a). However, in contrast to expectations, neither PF‐06409577 nor SBI‐0206965 altered the conversion of LC3 I to II in both Raw264.7 cells and BMDMs (Figure 5b,c).

To explore the underlying mechanism, oxidative stress was examined given that ROS generation increases during hypoglycaemia^29^ and has been reported to stimulate MIF release.^30^ Low glucose significantly elevated ROS levels in Raw264.7 cells (Figure 5d). Despite the sizeable reduction in MIF release following PF‐06409577 treatment, this compound did not alter the low glucose‐induced ROS production (Figure 5d). Interestingly, SBI‐0206965 significantly increased ROS levels (Figure 5e). However, further examination revealed this to be AMPK independent, given that the compound increased MIF release from AMPKα1/α2 KO MEF cells in cell apoptosis‐dependent manner (ESM Figure 6).

### 3.4 Low glucose‐induced MIF release was NFĸB dependent

**FIGURE 6:**
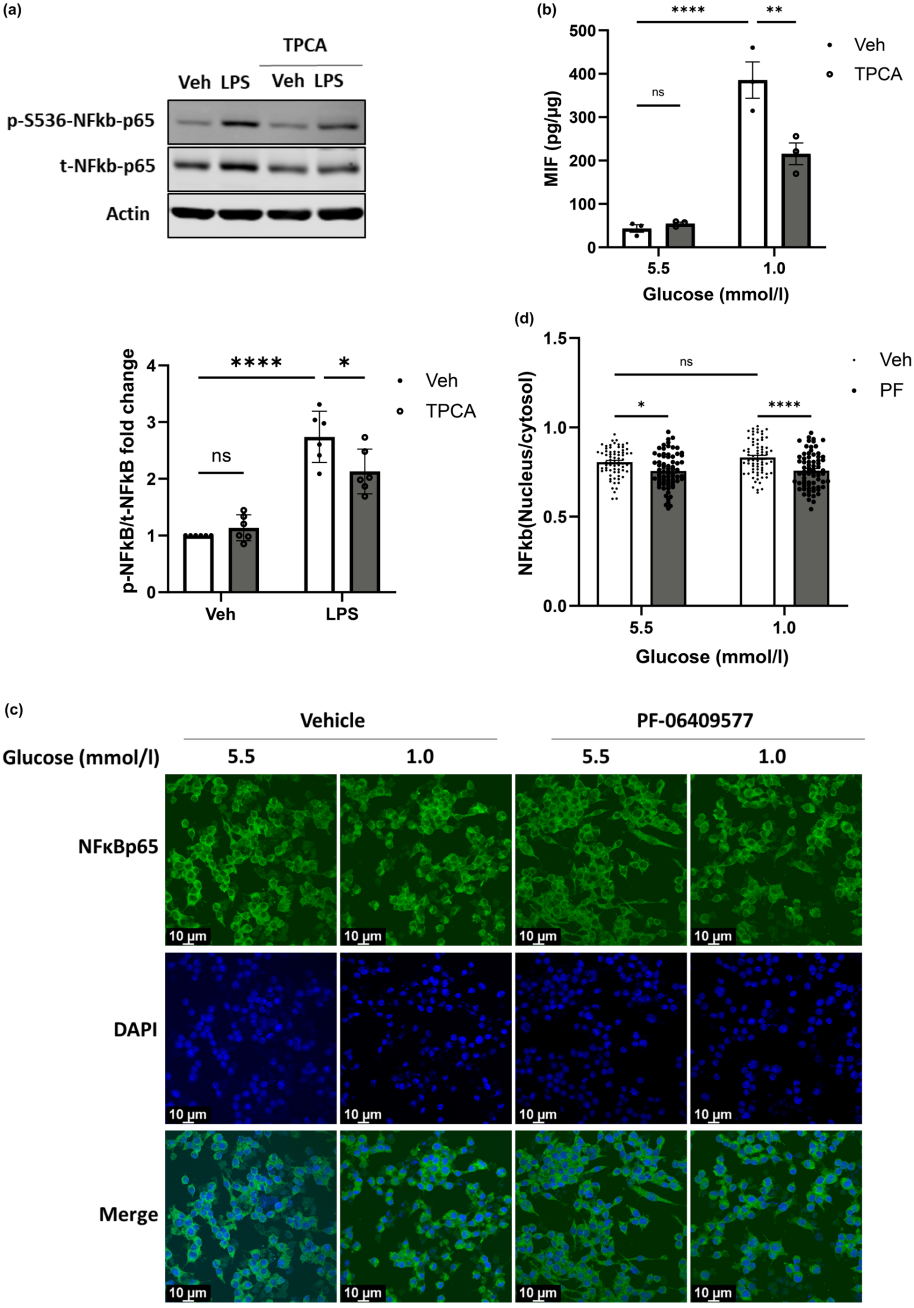
PF‐06409577 reduced the translocation of NFĸBp65 and TPCA inhibited LPS‐induced NFĸB activation and low glucose‐induced MIF release in Raw264.7 cells. (a) Representative immunoblots of Raw264.7 cells exposed to vehicle (0.1% v/v DMSO) (Veh) or 100 ng/mL of LPS in the presence of 1 μmol/L of TPCA for 6 h (*n* = 6). Densitometric analysis of immunostaining for phosphorylated protein was normalised to total protein level. p‐NFĸB, phospho‐S536‐ NFĸB‐p65 (p‐S536‐ NFĸB‐p65); t‐NFĸB indicates total NFĸB‐p65 (t‐NFĸB). (b) Raw264.7 cells were cultured with either 5.5 mmol/L or 1.0 mmol/L glucose for 16 h and in the presence of 1 μmol/L of TPCA for the last 6 h, MIF released in media was assessed in ELISA (*n* = 3). (c) Raw264.7 cells seeded on coverslips were cultured with either 5.5 mmol/L or 1.0 mmol/L glucose for 16 h and in the presence of 10 μmol/L of PF‐06409577 for 16 h followed by immunocytochemistry staining with NFĸBp65 as Alexa fluor488 and nucleus with DAPI (*n* = 3). (d) The green fluorescence intensity in the nucleus and cytosol was measured in FIJI, and data were collected from three independent experiments, 24 cells from four separate images in each experiment (*n* = 72). Data are expressed as mean ± SEM. Comparisons between groups were made by two‐way ANOVA with Bonferroni's multiple comparisons test. *p < 0.05; **p < 0.01; ****p < 0.0001; ns, not significantly different.

AMPK can inhibit and/or reduce translocation of NFĸB to the nucleus,^31^ to reduce pro‐inflammatory cytokine production. Here, TPCA‐1 was used to inhibit NFĸB,^32^ with lipopolysaccharide (LPS) used as a positive control.^33^ LPS‐induced phosphorylation of NFĸB was blocked by TPCA‐1 in Raw264.7 cells, as expected (Figure 6a), and importantly, TPCA‐1 also attenuated low glucose‐induced MIF release (Figure 6b), suggesting that NFĸB plays a role in low glucose‐induced MIF secretion. There was a modest, non‐significant trend towards increased phosphorylated‐NFĸB‐p65 after low glucose exposure in Raw264.7 cells that was not observed in BMDMs (ESM Figure 7). Moreover, PF‐06409577 did not significantly alter NFĸB phosphorylation; however, it did significantly reduce, albeit modestly, the nuclear/cytoplasmic ratio of NFĸB (Figure 6d).

## 4 DISCUSSION

Our studies demonstrate for the first time low glucose‐induced MIF release from primary BMDMs, Raw264.7 cells and BMDM cells isolated from AMPK gain‐of‐function mice. This is a key finding as previous studies have demonstrated an association between circulating MIF and adipose tissue inflammation, development of insulin resistance, β‐cell dysfunction and atherosclerosis.^34^ Linked to this, MIF levels correlate with the progression to atherosclerosis,^35^, ^36^ and severe hypoglycaemia is a risk factor for an atherosclerotic cardiovascular event.^37^ Taken together, these data suggest that AMPK activation using next‐generation allosteric AMPK activators could be useful for reducing inflammation and hence cardiovascular risk.

In our study, we observed no change in the cellular MIF expression in macrophages. However, consistent with the finding in adipocytes,^38^ we found that low glucose elevated MIF secretion. Currently, the mechanisms of MIF release are poorly defined though cell death and loss of autophagy likely contribute to MIF release.^30^ We ruled out cell death in our studies, at least over the time course studied. MIF is constitutively expressed and contained within readily releasable pools in contrast to other pro‐inflammatory cytokines and suggests that MIF may act as first responder during hypoglycaemia.^39^ In contrast to expectations, we did not observe changes in other pro‐inflammatory cytokines such as TNFα and IL‐1 β release from unpolarised macrophages. It will be interesting to determine in future studies whether low glucose generates a greater and broader increase in pro‐inflammatory cytokine production from polarised macrophages, from macrophages isolated from models of diabetes/people with diabetes or during glucose recovery following a hypoglycaemia‐like challenge. Polarised pro‐inflammatory M1 macrophages switch to aerobic glycolysis, and this metabolic switch is associated with reduced AMPK activation,^15^ whereas anti‐inflammatory M2 macrophages utilise mainly oxidative metabolism to generate ATP. In our naïve macrophages, we did not see a metabolic shift towards glycolysis, and in contrast, low glucose decreased the ratio of glycolysis to oxidative phosphorylation. This effect persisted for up to 3 h, suggesting a successful metabolic adaptation to preserve intracellular ATP levels. Interestingly, pharmacological AMPK activation inhibited or reduced the necessity for this metabolic adaptation to low glucose. This begs the question as to what, if any, is the mechanistic difference between physiological (low glucose) and pharmacological AMPK activation? AMPK pathway activity modestly increased in response to low glucose and this coincided with increased MIF release. We tried to examine whether AMPK inhibition would further augment MIF release; however, SBI‐0206595 increased MIF release in an AMPK‐independent manner, mostly likely by increasing ROS production. Therefore, we cannot say that this modest physiological AMPK activation is restraining MIF release. However, BMDMs isolated from AMPK D316A allowed us to examine non‐pharmacological effects. In line with the pharmacology studies, modest genetic AMPK activation attenuated low glucose‐induced MIF release. These data lend support to the idea that increasing levels of AMPK activity produce greater suppression of MIF release and that the regulation of MIF by AMPK is most apparent when there is an energetic stress to work against.

AMPK is generally regarded as a negative regulator of pro‐inflammatory cascades, although the underlying mechanism is still unclear.^40^ AMPK in haematopoietic cells reduces mouse adipose tissue macrophage inflammation and insulin resistance.^41^ Our data demonstrate that pharmacological AMPK activation, using two different AMPK activators, attenuates MIF release from macrophages. Previous studies have shown that hypoglycaemia/low glucose induces oxidative stress,^10^ and separate studies have shown that AMPK can inhibit oxidative stress.^42^, ^43^ There is also evidence that oxidative stress induces MIF release.^44^ Our data suggest that low glucose‐mediated oxidative stress may drive MIF release. However, PF‐06409577 did not alter low glucose‐induced ROS production, suggesting that AMPK‐mediated suppression of MIF occurs further downstream.

Surprisingly, we found no evidence that PF‐06409577 altered autophagy. Despite a recent report demonstrating PF‐06409577‐mediated reductions in the ratio of LC3 I/II in osteosarcoma cells,^45^ this was not observed in macrophages even at higher concentrations (10 μmol/L vs. 1 μmol/L). Collectively, our findings suggest that low glucose‐induced MIF release is likely mediated, at least in part by increasing oxidative stress and that AMPK acts downstream of oxidative stress to suppress MIF release. The regulation of MIF secretion is poorly understood. Unlike most other cytokines, MIF lacks the N‐terminal signal peptide that guides secretion through the conventional endoplasmic reticulum (ER)‐Golgi route; therefore, it is secreted via an unconventional pathway.^46^ Previous studies demonstrated that the inhibition of autophagy using RNAi or autophagy inhibitor, 3‐methyladenine (3‐MA) enhanced MIF release from macrophages.^30^ Consistently, we observed that AMPK/ULK/autophagy inhibitor SBI‐0206965 substantially enhanced MIF release from macrophages, despite no effect on LC3 conversion. In the present study, low glucose‐induced autophagy coincided with increased MIF release, indicating that autophagy is not directly involved in the regulation of MIF secretion from macrophages. Here, it is likely that the suppression of MIF occurs through an AMPK‐NFĸB signalling pathway given the role of NFĸB in the anti‐inflammatory actions of AMPK in other pathological states.^47^ AMPK inhibits the translocation of NFĸB where it can otherwise activate transcription of genes involved in pro‐inflammatory signalling^33^ and we confirmed this in the present study, although this effect was modest. Similarly, NFĸB inhibition attenuated low glucose‐induced MIF secretion, further demonstrating that NFĸB is involved in MIF release. However, given that we did not find production of other pro‐inflammatory cytokines in our studies, it is plausible that MIF is acting in a protective and or compensatory manner to help cells defend against low energy availability.

## 5 CONCLUSION

In summary, these data suggest that macrophage AMPK acts to limit MIF release under pathological states where increased ROS‐mediated activation of NFĸB occurs. Moreover, pharmacological AMPK activation suppresses MIF release without altering the generation of ROS, at least in response to low glucose. Taken together, this suggests that pharmacological AMPK activation could be a strategy for reducing macrophage MIF release in diseases such as diabetes, where glucose variation frequently occurs. Further study is required to determine the regulation and downstream action of MIF signalling in the context of low glucose.

## CONFLICTS OF INTEREST

The authors have no conflicts of interest to declare.

## FUNDING INFORMATION

This work was supported in part by grant MR/N0137941/1 for the GW4 BIOMED MRC DTP, awarded to the Universities of Bath, Bristol, Cardiff and Exeter from the Medical Research Council (MRC)/UKRI, which supported J.Z. and E.P.; a Diabetes UK RD Lawrence Fellowship (13/0004647) to C.B. A.P was supported by an AstraZeneca postdoctoral fellowship and a BBSRC Discovery Fellowship (BB/W009633/1). D.C. was supported by the Medical Research Council (MC‐A654‐5QB10). Work was undertaken in the Biological Services Unit at University of Exeter and Imperial College London. The views expressed are those of the authors and not necessarily those of the NIHR or Department of Health and Social Care. For the purpose of open access, the author has applied a ‘Creative Commons Attribution’ (CC BY) licence to any Author Accepted Manuscript version arising.

## Supporting information


Data S1.

## Data Availability

All data generated or analysed during this study are included in the published article (and its ESM). The file is available from the corresponding author upon reasonable request.
