# Supplementary material for: Hypoglycaemic stimulation of macrophage cytokine release is suppressed by AMP‐activated protein kinase activation
Source: Diabet Med. 2024 Dec 24;42(3):e15456. doi: 10.1111/dme.15456 (PMC11823358; doi:10.1111/dme.15456)
Supplement: Supplementary file 1 — Data S1. [file DME-42-e15456-s001.zip › ESM Figures.pdf]

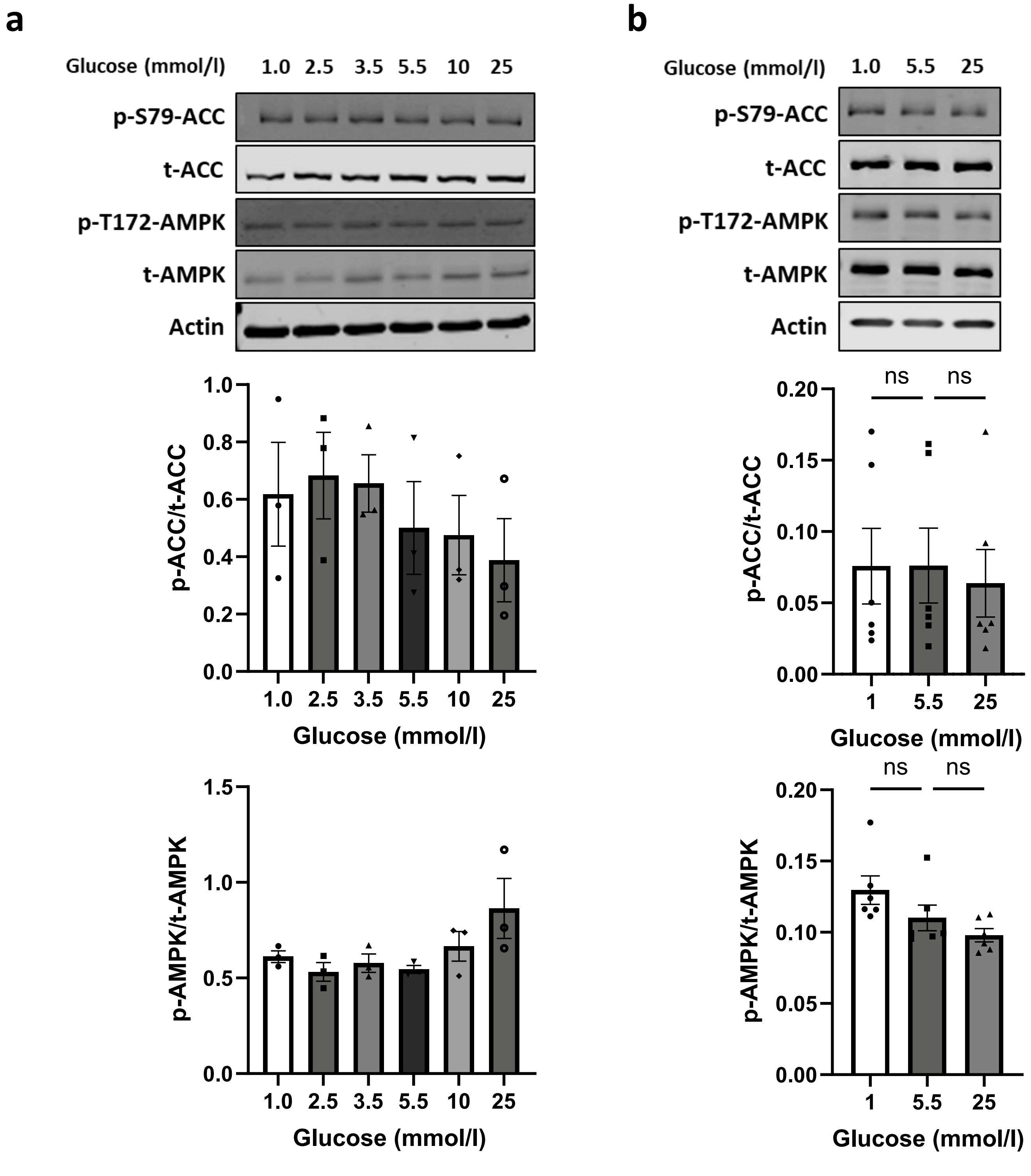

Supplementary Figure 1 The effect of different concentrations of glucose on AMPK activation in Raw264.7 cells and BMDMs

a) Raw254.7 cells (n = 3) and **b)** BMDMs were cultured with different concentrations of glucose for 16 hours (n = 6). Cells were then lysed, and immunoblots were prepared. Densitometric analysis of immunostaining for phosphorylated protein was normalised to total protein level. p-ACC, phospho-acetyl-CoA caboxylase (S79) (p-S79-ACC); p-AMPK, phospho-AMP-activated protein kinase (T172)(p-T172-AMPK); t-ACC indicates total acetyl-CoA carboxylase; t-AMPK, total AMP-activated protein kinase. Data are expressed as mean±SEM. Comparisons between groups were made by one-way ANOVA with Bonferroni's multiple comparisons test. ns, not significantly differnt.

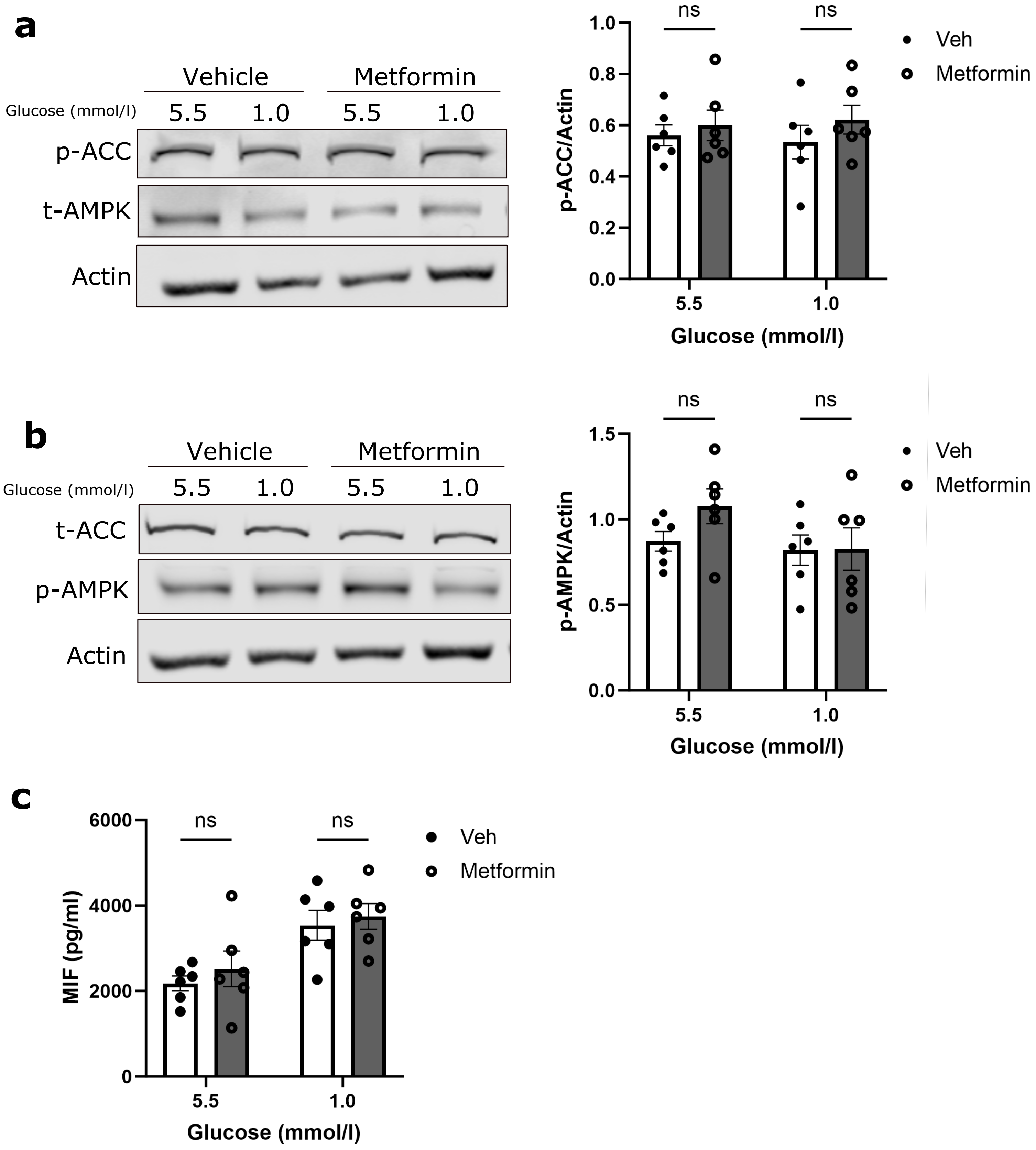

Supplementary Figure 2 Application of metformin had no effect on AMPK activation status or MIF release from Raw264.7 cells.
a,b) Raw264.7 cells were cultured in different concentrations of glucose with either vehicle (sterile water) or 250μmol/l metformin for 16 hours. The cells were then lysed and immunoblots prepared. Densitometric analysis of immunostaining for phosphorylated protein was normalised to actin. p-ACC, phospho-acetyl-CoA carboxylase (S79); t-ACC, total acetyl-CoA carboxylase; p-AMPK, phospho-AMP-activated protein kinase (T172); t-AMPK, total AMP-activated protein kinase. **c)** Supernatant was collected from the cells **(a,b)** and the concentration of MIF present was assessed by ELISA. Data are expressed as mean±SEM (n=6).

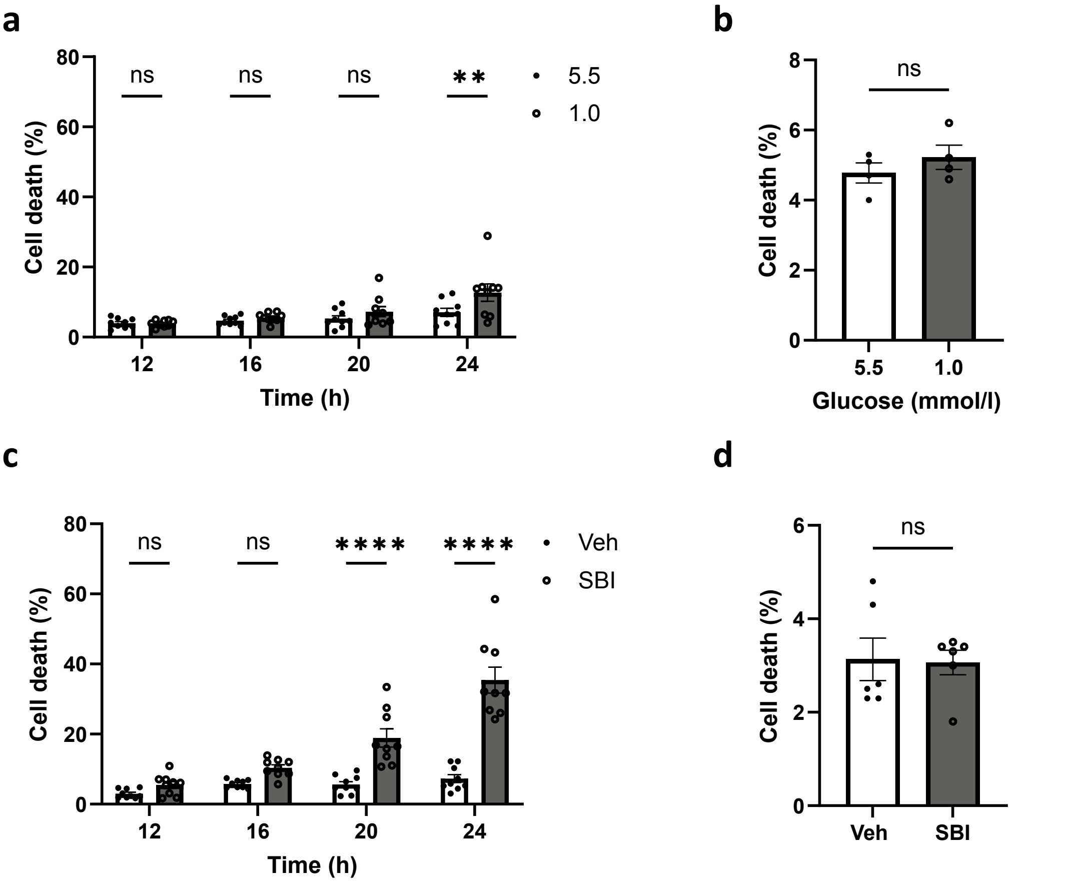

Supplementary Figure 3 Low level of glucose (1.0 mmol/l) and SBI-0206965 increased cell death over time in Raw264.7 cells, but no significant cell death were detected at 16 hours for either Raw264.7 cells or BMDMs.

a) The time course of cell death of Raw264.7 cells cultured with 5.5 mmol/l or 1.0 mmol/l glucose for up to 24 hours (n = 9). **b)** The cell death of BMDMs exposed to 5.5 mmol/l or 1.0 mmol/l glucose for 16 hours (n = 3). **c)** The time course of cell death of Raw264.7 cells exposed to vehicle (0.1% v/v DMSO) (Veh) or 30 µmol/l of SBI-0206965 (SBI) for up to 24 hours (n = 9(. **d)** The cell death of BMDMs exposed vehicle (0.1% v/v DMSO) (Veh) or 30 µmol/l of SBI-0206965 (SBI) for 16 hours (n = 6). Cells viability was assessed at indicated time points by staining with propidium iodide followed by flowcytometry. The numbers of postive staining cells were expressed as percentage of total cell numbers. Data are expressed as mean±SEM. Comparisons between groups were made by two-way ANOVA with Bonferroni's multiple comparisons test **(a, c)** or unpaired t test **(b, d)**. **P<0.01; ns, not significantly different.

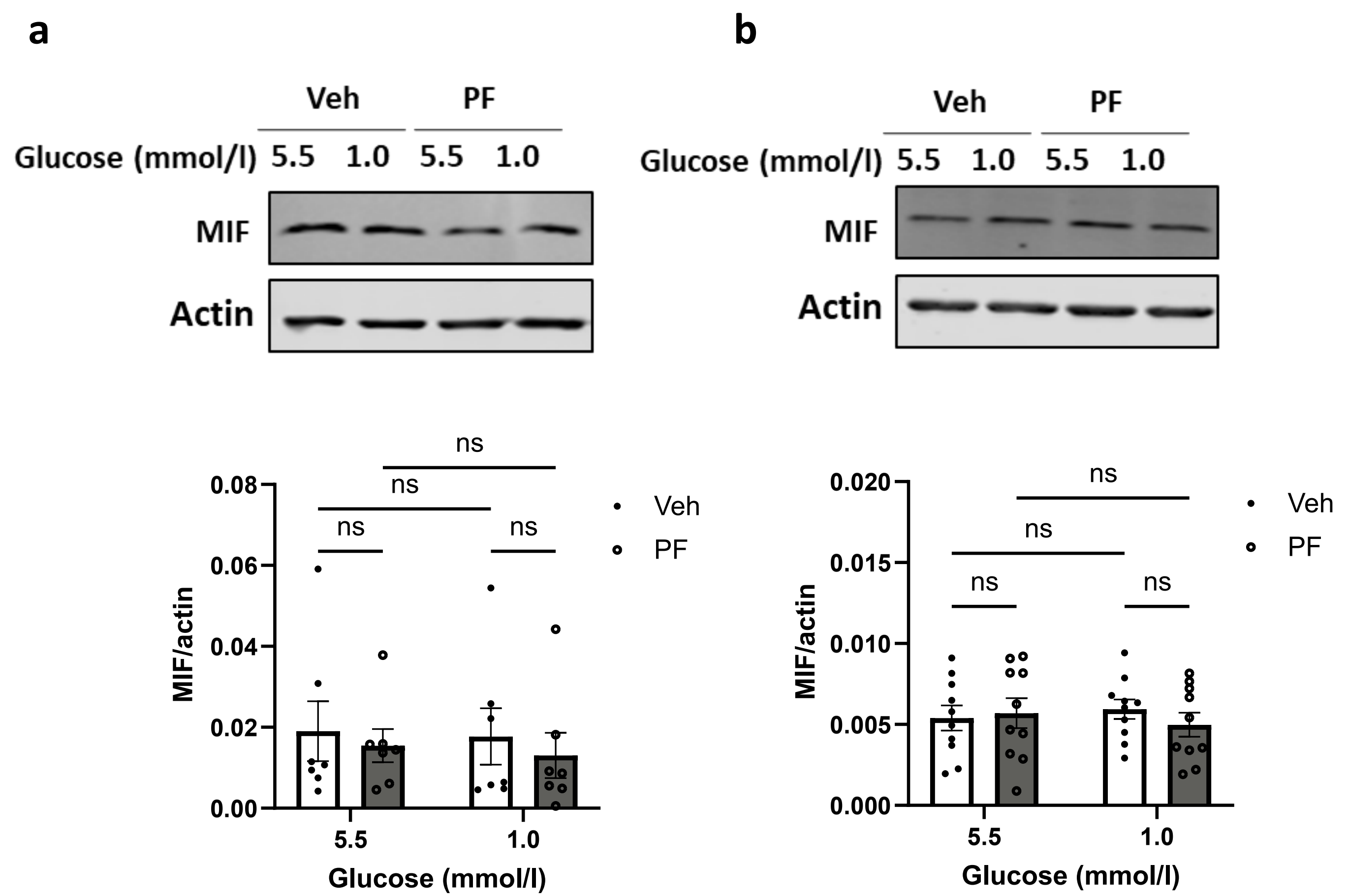

Supplementary Figure 4 low glucose and/or PF-06409577 did not alter the intracellular expression of MIF in macrophages

a) Raw264.7 cells (n = 7) and **b**) BMDMs were cultured with either 5.5 mmol/l or 1.0 mmol/l of glucose in the presence of vehicle (0.1% v/v DMSO) (Veh) or 10 µmol/l of PF-06409577 (PF) for 16 hours (n = 10). Cells were then lysed, and immunoblots were prepared. Densitometric analysis of immunostaining for MIF was normalised to actin, Data are expressed as mean±SEM. Comparisons between groups were made by two-way ANOVA test. ns, not significantly different.

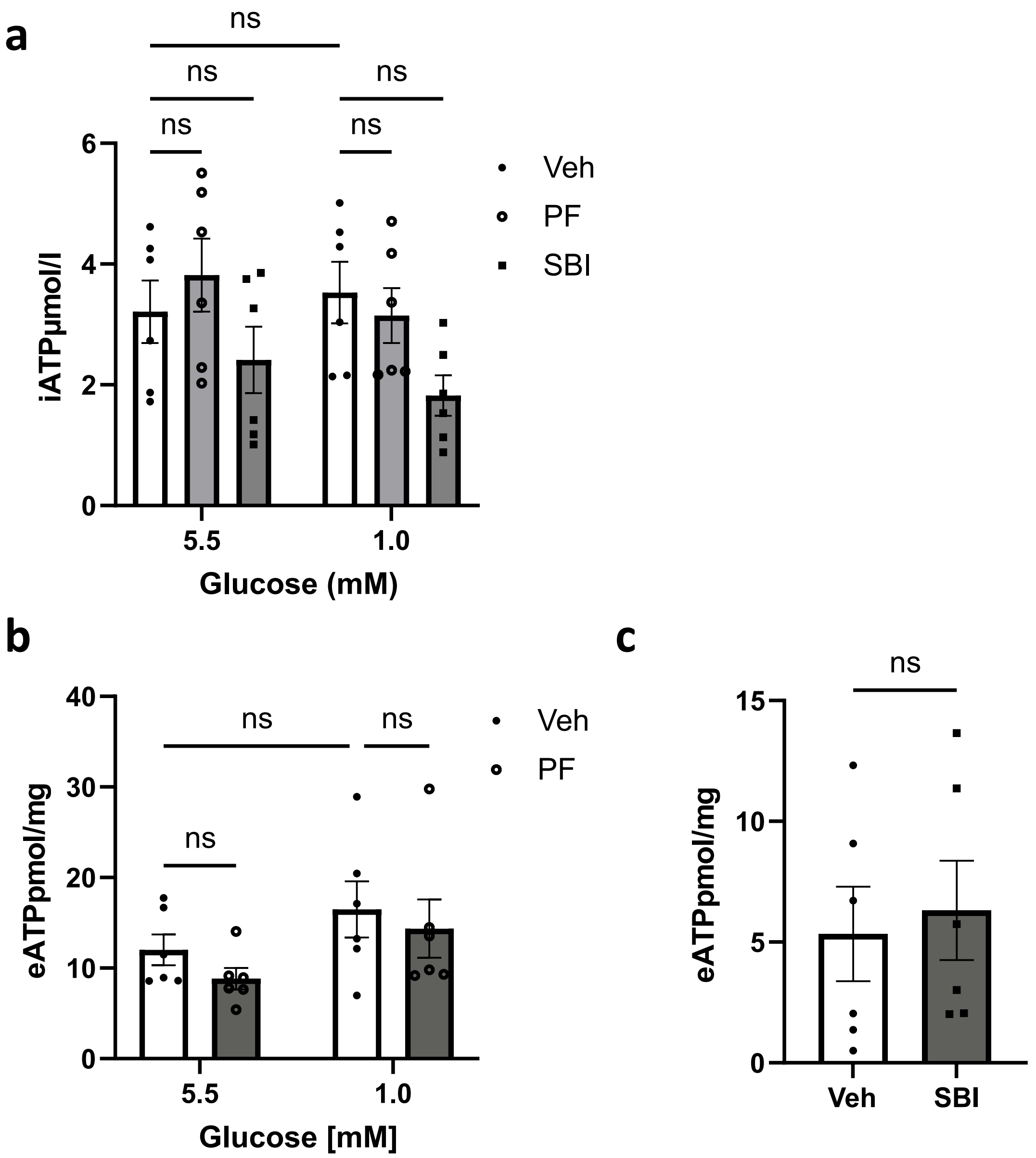

Supplementary Figure 5 Low glucose (1.0 mmol/l), PF-06409577 or SBI-0206965 did not alter the levels of iATP or eATP in Raw264.7 cells

a) Raw264.7 cells were cultured in the presence of 5.5 mmol/l or 1.0 mmol/l of glucose with vehicle (0.1% v/v DMSO) (Veh) or 10 µmol/l of PF-06409577 (PF) or 30 µmol/l of SBI-0206965 (SBI) for 16 hours. The cells in the well were then lysed and intracellular ATP (iATP) were measured using luminescence based assay ATPlite. **b**) The extracellular ATP (eATP) released in the media was measured using luminescence based assay ATPlite and normalised to total protein content in the same dishes (**b, c**). Data are expressed as mean± SEM (n=6), Comparisons between groups were made by two-way ANOVA with Bonferroni's comparison test (**a, b**), or unpaired t test (**c**). ns, not significantly different.

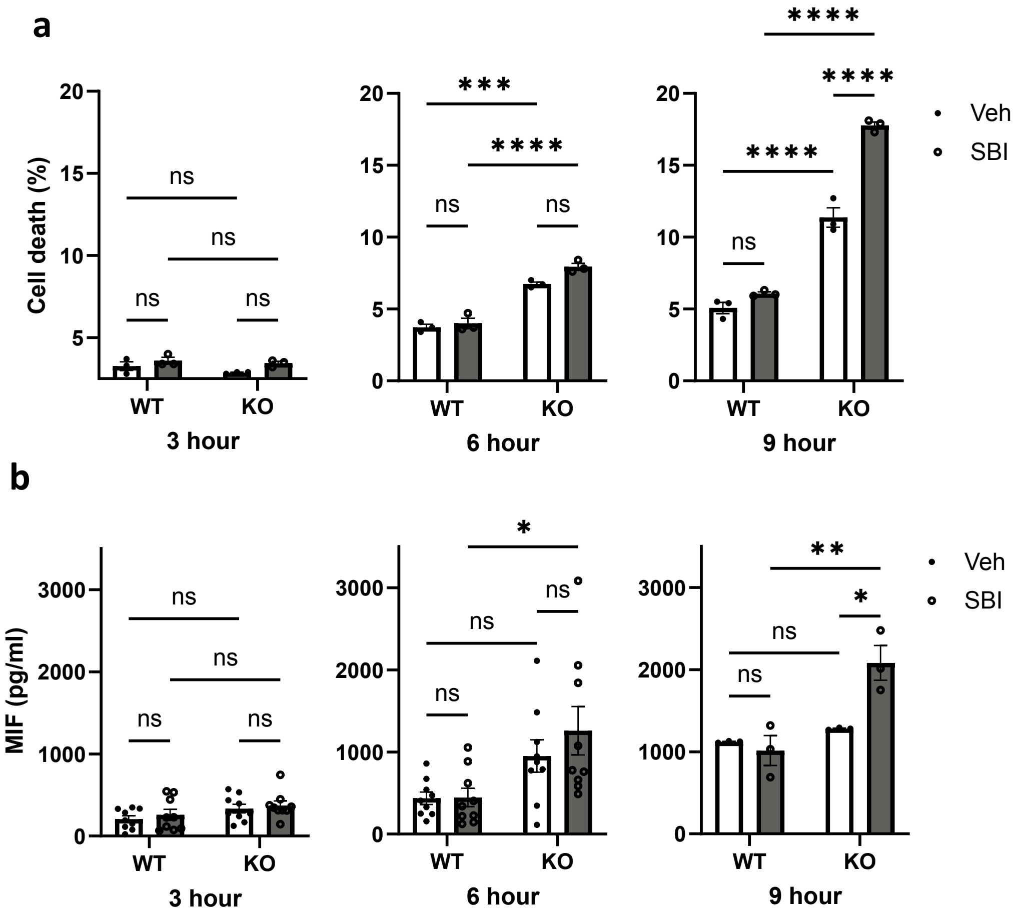

Supplementary Figure 6 The cell death of AMPK α1/2 (-/-) MEFs was elevated after 6 hours and SBI-0206965 induced cell death and MIF release from AMPK α1/2 (-/-) MEFs at 9 hours

a) Wild-type (W) and AMPK α1/2 (-/-) knock out (KO) MEF cells wer were cultured with vehicle (0.02% v/v DMSO) (Veh), or 10 μmol/l of SBI-0206965 (SBI) for 3 hours, 6 hours, and 9 hours. Cells viability was assessed by staining with propidium iodide followed by flowcytometry. The numbers of postive staining cells were expressed as percentage of total cell numbers (n = 3). **b**) The MIF levels in medium was assessed by ELISA (n = 9 for 3 and 6 h, n = 3 for 9 h). Data are expressed as mean±SEM. Comparisons between groups were made by two-way ANOVA with Bonferroni's multiple comparisons test. *P<0.05; **P<0.01; ***P<0.001; ****P<0.0001; ns, not significantly different.

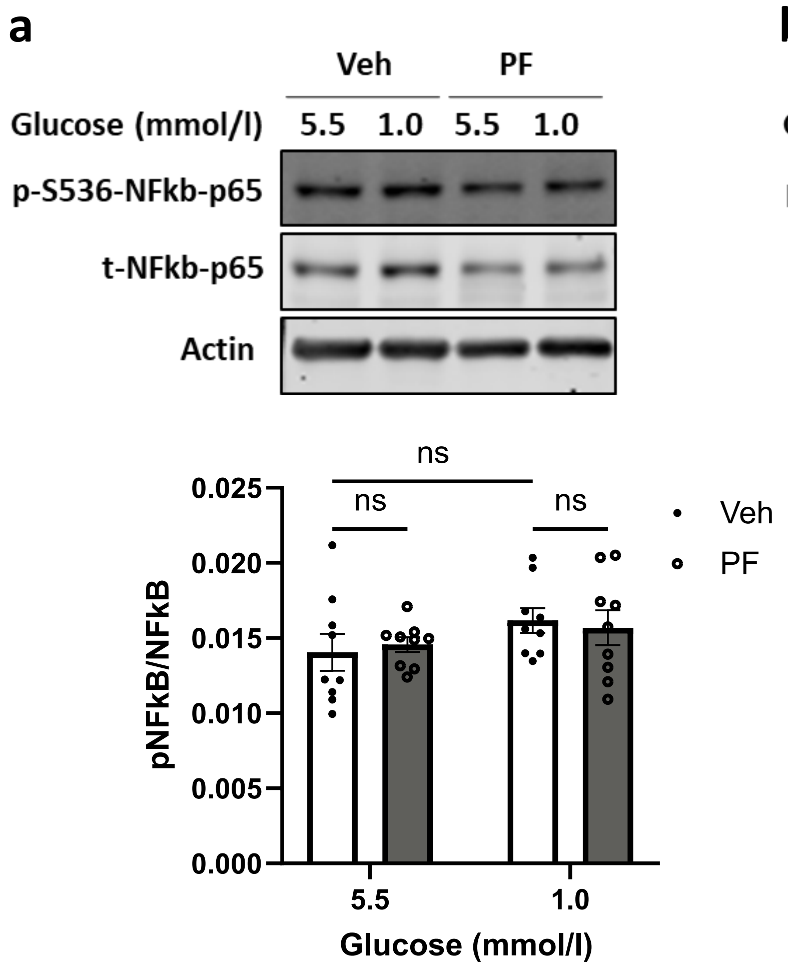

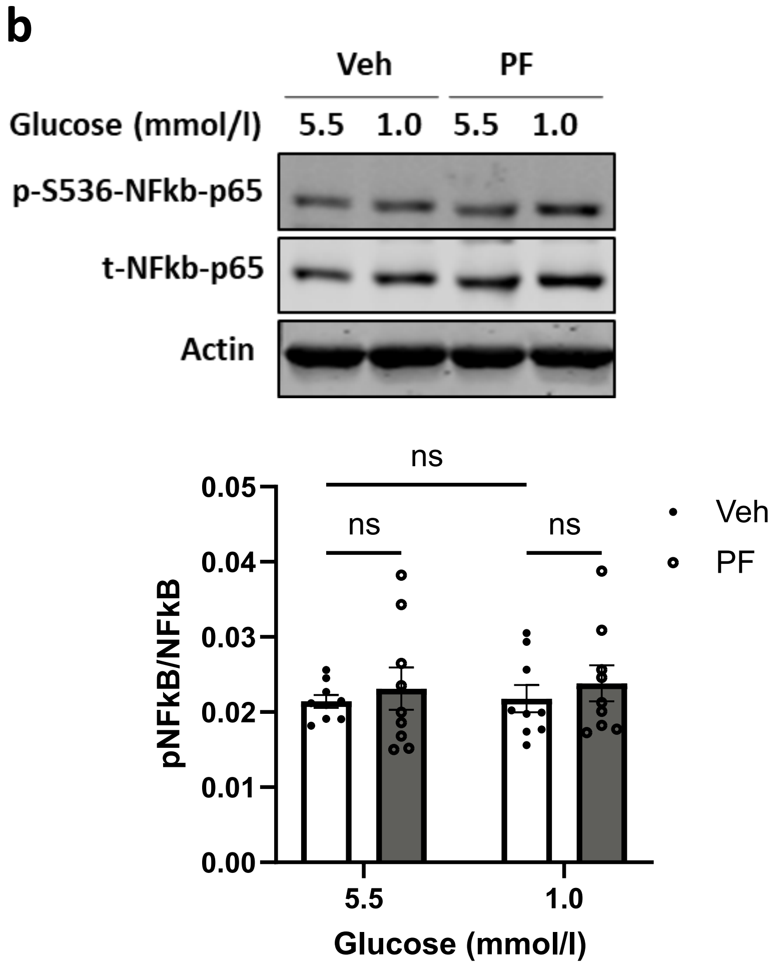

Sup Figure 7 Low glucose or PF-06409577 did not alter the level of phosphorylation of NFκB-p65 in Raw264.7 cells or BMDMs

a) Raw 264.7 cells (n = 9(or **b)** BMDM cells were cultured with either 5.5 mmol/l or 1.0 mmol/l of glucose in the presence of vehicle (0.1% v/v DMSO) (Veh) or 10 µmol/l of PF-06409577 (PF) for 16 hours (n = 9). Cells were then lysed, and immunoblots were prepared. Densitometric analysis of immunostaining for phosphorylated protein was normalised to total protein level. p-S536-NFκB-p65 indicates phosphoylated NFκB-p65 at S536; t-NFκB-p65 indicates total NFκB-p65. Data are expressed as mean±SEM. Comparisons between groups were made by two-way ANOVA with Bonferroni's multiple comparisons test. ns, not significantly different.
