# Supplementary material for: Hypoglycaemic stimulation of macrophage cytokine release is suppressed by AMP‐activated protein kinase activation
Source: Diabet Med. 2024 Dec 24;42(3):e15456. doi: 10.1111/dme.15456 (PMC11823358; doi:10.1111/dme.15456)
Supplement: Supplementary file 1 — Data S1. [file DME-42-e15456-s001.zip › ESM Methods Final.docx]

***Cell culture***

Cell were culture in DMEM medium (Sigma-Aldrich D5671; 25 mmol/l glucose, containing 10% FBS, 1% Pen/Strep v/v, 4 mmol/l Glutamine) and were maintained in humidified incubators with 95% O^2^/5% CO^2^. Primary bone marrow-derived macrophages (BMDMs) were generated as previously described (*30*). Briefly, bone marrow was flushed out from the femur and tibia of 9- to 11-week-old C57BL/6J mice and was differentiated in flasks for 8 days in BMDM culture medium: RPMI (RPMI 1640; Gibco 21875-059, 11 mmol/l glucose) with 20% heat-inactivated FBS, 20% L929s conditional medium as a source of M-CSF and 2% penicillin/streptomycin. On day 9, cells were harvested and frozen in liquid nitrogen. For experiments cell were plated in 60 mm dishes for 24 hours before treatment.

***Immunocytochemistry***

Immunocytochemical staining were performed as previously described (*35*). Raw264.7 cells were plated on 13 mm diameter glass coverslips. After treatment, cell were fixed and permeabilized in cold methanol for 90 seconds following by washing with PBS-T (0.3% vol/vol of triton in PBS). Non-specific binding was blocked with donkey serum (5% vol/vol in PBS-T; 15 mins) before incubating with mouse anti-NFĸBp65 (1:1000 in TBST) overnight at 4 °C. Sequentially, incubated with fluorescent secondary donkey anti-mouse Alexa fluor488 (1:500 in PBS-T) for 1 hour following with washing step. A secondary antibody control was also performed. Coverslips were then mounted in mounting medium with DAPI (abcam) and allowed to dry prior to imaging using Leica con-focal microscopy. FIJI cell counter was used to manually analyse fluorescent staining.

***Measurement of extracellular and intracellular ATP levels***

ATP levels were measured using ATPlite assay kit (no. 6016941, Perkin Elmer, Seer Green, UK). Briefly, 100 µl of media supernatant was used per sample, in black-walled 96 well plates according to manufacturer’s instructions. The luminescence was read using the PheraStar microplate reader. ATP concentrations were calculated and normalised to total protein assessed from lysates from the same experiment. The same methodology was used to measure the levels of intracellular ATP. Raw264.7 cells were plated in black-walled 96 well plates 24 hours before treatments then cells were lysed and luminescence was read and intracellular ATP levels was presented as pmol/l.

***Cell viability assay***

Cell viability assay was performed by propidium iodide (PI) staining followed with flowcytometry analysis (*35*). Briefly, cells were seeded in 12-well plates for treatments, at indicated time, cells were dissociated with 0.05% w/v trypsin-EDTA (combined with scraping for Raw264.7 cells) and all the cells were collected and pelleted by centrifuging followed by re-suspending in 200 µl of DMEM or RPMI medium and staining with 200 µl of propidium iodide (2 µg/ml) in fluorescence activated cell sorting (FACS) buffer (2% v/v FBS in PBS) for 10 minutes. Subsequently, stained cells was determined using flow cytometry (BD Accuri C6; BD Systems; UK). The events with high fluorescence relative to cell size as determined using the 488 nm laser combined with FSC were classified as dead cells and expressed as the percentage of total cell numbers.

**Western blotting**

Around 2x10^6^ Raw264.7 cells were seeded onto 60 mm petri dishes one day before treatment and harvested in 65µL lysis buffer. Protein concentrations were assessed via Bradford assay, and 10µg protein from each sample was loaded per gel (Merck mPAGE® 4-12% Bis-Tris Precast Gels). Proteins were transferred to nitrocellulose membranes and blocked with 5% milk (wt/vol) in TBS/T. Membranes were then probed with antibodies against target proteins (phospho-acetyl-CoA carboxylase (S79) rabbit antibody, Cell Signalling Technologies 3661S; acetyl-CoA carboxylase mouse antibody, Millipore 05-1098; phospho-AMPK (T172) rabbit antibody, Cell Signalling Technologies 2535L; AMPKα (F6) mouse antibody, Cell Signalling Technologies 2793S; β-actin rabbit antibody, Cell Signalling Technologies 4967S; all diluted 1:1000 in 2.5% (wt/vol) BSA-TBS/T and incubated with membranes overnight at 4^o^C). Membranes were then incubated with the appropriate secondary antibodies (IRDye 680RD Donkey anti-Mouse, LI-COR 926-68072; IRDye 800CW Donkey anti-Rabbit, LI-COR 926-32213; both diluted 1:10,000 in TBS/T) for 2 hours at room temperature. Images were taken using infrared imaging and changes in expression quantified by densitometry. Changes in phosphorylation of target proteins were normalised to actin expression.
