# Supplementary material for: Hypoglycaemic stimulation of macrophage cytokine release is suppressed by AMP‐activated protein kinase activation
Source: Diabet Med. 2024 Dec 24;42(3):e15456. doi: 10.1111/dme.15456 (PMC11823358; doi:10.1111/dme.15456)
Supplement: Supplementary file 1 — Data S1. [file DME-42-e15456-s001.zip › Sup fig 7-DM-13Sept24.pdf]

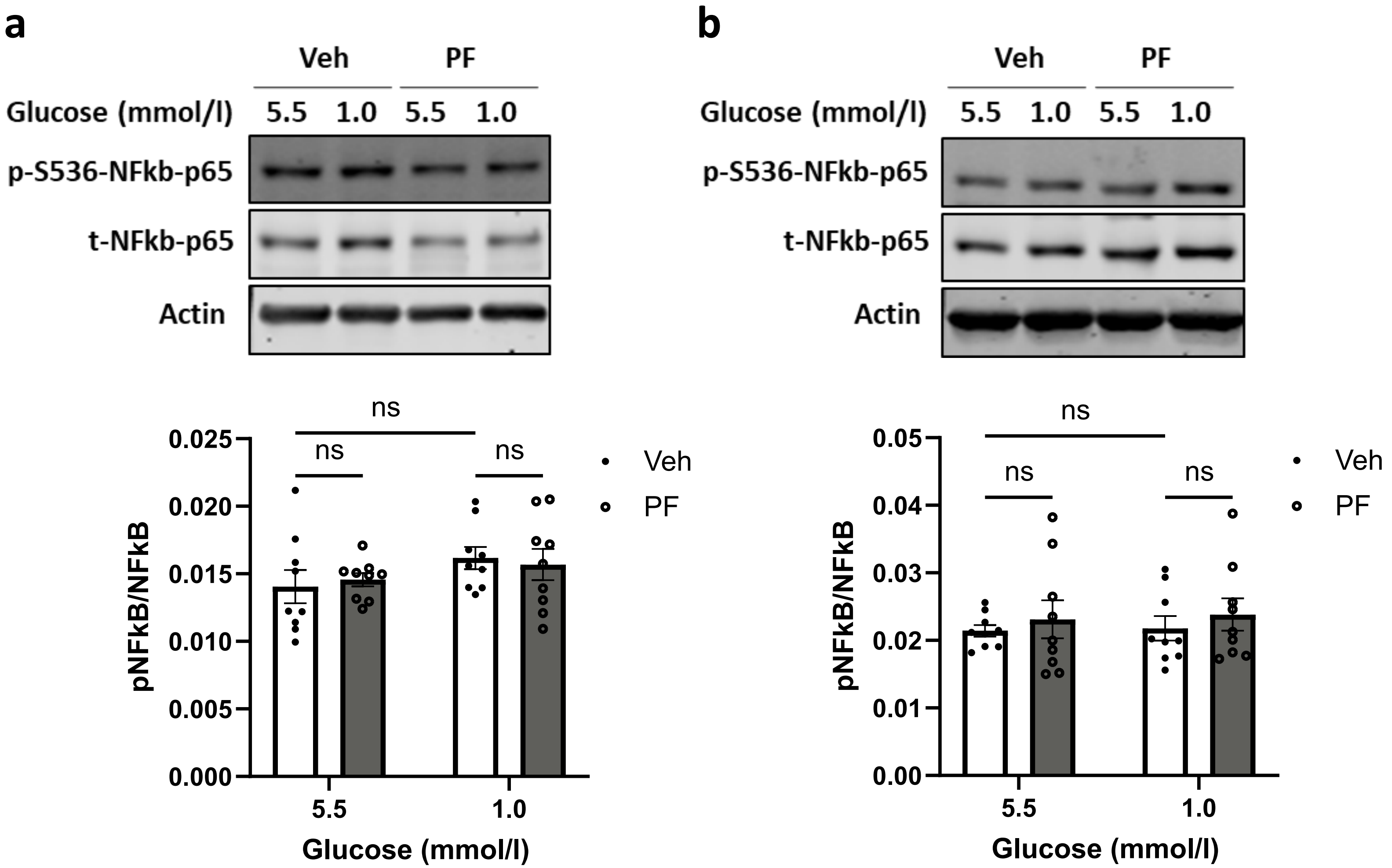

Sup Figure 7 Low glucose or PF-06409577 did not alter the level of phosphorylation of NFκB-p65 in Raw264.7 cells or BMDMs

a) Raw 264.7 cells (n = 9(or **b)** BMDM cells were cultured with either 5.5 mmol/l or 1.0 mmol/l of glucose in the presence of vehicle (0.1% v/v DMSO) (Veh) or 10 µmol/l of PF-06409577 (PF) for 16 hours (n = 9). Cells were then lysed, and immunoblots were prepared. Densitometric analysis of immunostaining for phosphorylated protein was normalised to total protein level. p-S536-NFκB-p65 indicates phosphoylated NFκB-p65 at S536; t-NFκB-p65 indicates total NFκB-p65. Data are expressed as mean±SEM. Comparisons between groups were made by two-way ANOVA with Bonferroni's multiple comparisons test. ns, not significantly different.
